# Biomaterial Fg/P(LLA-CL) regulates macrophage polarization and recruitment of mesenchymal stem cells after endometrial injury

**DOI:** 10.1007/s10856-024-06807-w

**Published:** 2024-07-29

**Authors:** Sirui Song, Anfeng Wang, Siyu Wu, Huaifang Li, Hongbing He

**Affiliations:** 1 Department of Obstetrics and Gynecology, Tongji Hospital of Tongji University, School of Medicine, Tongji University, Shanghai, 200065 China; 2 Department of Obstetrics and Gynecology, Chengdu Women’s and Children’s Central Hospital, School of Medicine, University of Electronic Science and Technology of China, Chengdu, 610000 China; 3 Department of Gynecology and Obstetrics, Qilu Hospital (Qingdao), Cheeloo College of Medicine, Shandong University, Qingdao, 266000 China; 4 Shanghai Pine & Power Biotech Co. Ltd, Shanghai, 201108 China

## Abstract

The process of endometrial repair after injury involves the synergistic action of various cells including immune cells and stem cells. In this study, after combing Fibrinogen(Fg) with poly(L-lacticacid)-co-poly(ε-caprolactone)(P(LLA-CL)) by electrospinning, we placed Fg/P(LLA-CL) into the uterine cavity of endometrium-injured rats, and bioinformatic analysis revealed that Fg/P(LLA-CL) may affect inflammatory response and stem cell biological behavior. Therefore, we verified that Fg/P(LLA-CL) could inhibit the lipopolysaccharide (LPS)-stimulated macrophages from switching to the pro-inflammatory M1 phenotype in vitro. Moreover, in the rat model of endometrial injury, Fg/P(LLA-CL) effectively promoted the polarization of macrophages towards the anti-inflammatory M2 phenotype and enhanced the presence of mesenchymal stem cells at the injury site. Overall, Fg/P(LLA-CL) exhibits significant influence on macrophage polarization and stem cell behavior in endometrial injury, justifying further exploration for potential therapeutic applications in endometrial and other tissue injuries.

**Graphical Abstract:**

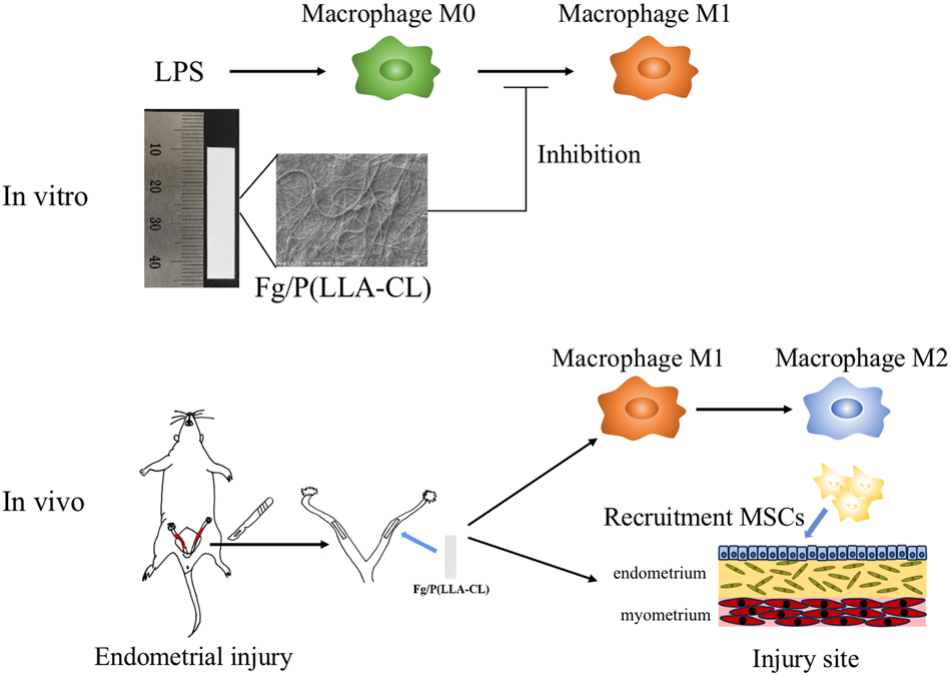

## Introduction

In China, the incidence of missed abortion is escalating annually, necessitating numerous women to undergo uterine procedures including curettage and hysteroscopy. Nevertheless, these procedures inevitably result in acute endometrial injury [1]. Metal instruments can potentially cause damage to the endometrial basal layer, leading to abnormal differentiation and a decrease in endometrial stem/progenitor cells. Post-operative chronic inflammation within the uterine cavity prompts immune cells, endometrial stromal cells, and epithelial cells to secrete a significant amount of collagen. This collagen accumulation ultimately results in the deposition of avascular fibrous tissues outside the cells, ultimately leading to the formation of intrauterine adhesions [2]. Intrauterine adhesions are characterized by symptoms such as amenorrhea, infertility, dysmenorrhea, and chronic pelvic pain, which significantly impact the quality of life and fertility of affected patients [3]. Due to the lack of effective treatment, absorbable biomaterials to promote endometrial repair have been increasingly explored.

Macrophages are crucial cells that play a pivotal role in inflammatory microenvironments and tissue remodeling processes. They are actively involved in every stage of tissue injury and repair, playing a leading role in restoring tissue homeostasis by eliminating cellular debris, remodeling the extracellular matrix (ECM), and synthesizing various cytokines and growth factors [4]. Following tissue injury, neutrophils are the first to arrive at the injury site, releasing cytokines that recruit macrophages. Subsequently, proinflammatory macrophages interact with effector T cells, forming a feedback loop that exacerbates the inflammatory response. Excessive expression of inflammatory factors can impair the regenerative capacity of tissue-resident stem cells/progenitor cells. In cases of severe endometrial injury, microbial imbalances within the endometrium result in increased lipopolysaccharide synthesis and sustained secretion of inflammatory cytokines by macrophages. This ultimately leads to excessive deposition of the ECM and endometrial fibrosis [5, 6]. Furthermore, macrophages constitute the central cell population in the response to biomaterials and the regulation of tissue regeneration, alternating between pro-inflammatory (M1) and anti-inflammatory (M2) phenotypes [7]. Modulating macrophage function can influence their capacity to recruit stem cells via paracrine signaling [8]. Consequently, the modulation of macrophages may represent a crucial approach for biomaterials to prevent intrauterine adhesions.

Fibrinogen is one of the most important proteins in the process of tissue remodeling after injury, which is involved in the regulation of coagulation and inflammation [9]. It has been previously believed that fibrinogen plays a pro-inflammatory role in multiple sclerosis, vascular wall disease, stroke, Alzheimer’s disease and rheumatoid arthritis [9]. However, recent studies have shown that fibrinogen can prevent LPS-stimulated macrophages from polarizing to a pro-inflammatory phenotype through NF-κB signaling pathway [10]. Furthermore, fibrinogen has the ability to regulate the recruitment of mesenchymal stem cells mediated by immune cells [11].

P(LLA-CL) exhibits complete biodegradability in vivo, decomposing into H_2_O and CO_2_. A wide range of cell types, including mesenchymal stem cells and fibroblasts, are capable of adhering to and proliferating on this material while maintaining their normal morphology [12]. In recent years, numerous tissue engineering materials have been developed utilizing P(LLA-CL) as a foundation. For instance, heparin-loaded P(LLA-CL) vascular scaffolds have demonstrated the ability to inhibit the activity of inflammatory smooth muscle cells, thereby reducing the incidence of stent stenosis and thrombosis [13]. Additionally, corneal endothelial cells loaded onto a transparent membrane composed of silk fibroin (SF) and P(LLA-CL) present a promising alternative for corneal transplantation in cases where donor tissue is limited [14]. Furthermore, nerve growth factor-encapsulated SF/P(LLA-CL) nanofibers have been shown to promote sciatic nerve regeneration in rats [15].

In our prior research, we successfully fabricated the nano-material Fg/P(LLA-CL) through electrospinning fibrinogen and P(LLA-CL) and subsequently confirmed the efficacy of Fg/P(LLA-CL) in facilitating tissue repair [16]. In this study, we examined the regulatory impact of Fg/P(LLA-CL) on macrophage function under inflammatory stimuli, both in vitro and in vivo in rats with endometrial injury. Additionally, we explored the capacity of Fg/P(LLA-CL) to recruit mesenchymal stem cells.

## Materials and methods

### Material preparation

The nanofibrous Fg/P(LLA-CL) film was fabricated, as previously described [16], by P&P Biotech Co. Ltd. (Shanghai, People’s Republic of China). In brief, fibrinogen was dissolved in hexafluoroisopropanol(HFIP), and P(LLA-CL) was also dissolved in the HFIP solution. The two solutions were stirred overnight and then mixed in a ratio of 1:4, and the total polymer concentration in the blend solution was maintained at 12 wt%. The prepared solution was put into a 5 ml needle tube and connected to an electrospinning device (Elmarco, The Czech Republic). The voltage was set to 20 kv, and the mixed solution was pumped at a speed of 0.8 ml/h to finally obtain a nanofiber membrane with a thickness of 0.2 mm. Rat adipose-derived mesenchymal stem cells (ADSCs) were obtained from Fenghui Biotechnology Co. Ltd (Changsha, People’s Republic of China) and cultured in DMEM medium (Biosharp, China) supplemented with 10% serum (Sigma, USA), 100 U/ml penicillin, and 0.1 mg/ml streptomycin (Biosharp, China). Subsequently, ADSCs were seeded onto Fg/P(LLA-CL) membranes at a density of 6 × 10^3^ cells per well in 24-well plates. The fiber diameter and cell adhesion state of the prepared nanomaterials were then examined using scanning electron microscopy.

### Macrophage polarization

Macrophages were induced from THP-1 cells purchased from ATCC (TIB-202, ATCC, USA). THP-1 cells were seeded at 5 × 10^5^ cells in six-well plates and cultured in RPMI 1640 medium (Biosharp, China) supplemented with 10% serum (sigma, USA), 100 U/ml Penicillin and 0.1 mg/ml Streptomycin (Biosharp, China). The THP-1 cells were induced into macrophages 48 h after adding Phorbol-12-myristate-13-acetate (PMA (beyotime, China))at a concentration of 100 ng/ml to the medium (Supplementary Fig. 1), then LPS (beyotime, China) at a concentration of 100 ng/ml was added to the culture medium to simulate inflammation and stimulate macrophages to polarize to M1 phenotype, with or without the addition of nanomaterial Fg/P(LLA-CL) extract. All experiments were performed in triplicate, the supernatant was collected after 48 h of incubation.

### Identification of macrophage polarization by immunofluorescence

Macrophages in different groups were fixed with 4% paraformaldehyde for 30 min and washed with PBS for 3 times, then permeabilized with Triton-X 100, and blockaded with BSA. The cells were then incubated with the appropriate primary antibodys (CD86/CD14,servicebio, China) for 6 h, followed by incubation with the appropriate fluorescent secondary antibodies (servicebio, China) for an additional 2 h. DAPI was added for 5 min to visualize the nuclei. Images were acquired using a fluorescence microscope.

### Quantitative real-time PCR

Total RNA was extracted from macrophages of different groups by RNA extraction kit(Absi, China). The extracted RNA was reverse-transcribed into cDNA using a one-step kit (Absin, China), and the target gene expression was detected using a real-time fluorescence quantitative PCR system (Bio-Rad, Maastricht, The Netherlands) after the reaction system was prepared using a RealUniversal Color PreMix (SYBR Green) kit (TIANGEN, China). The reaction primers were as follows:GAPDH: F 5′-AACGGATTTGGTCGTATTG-3′ R 5′-GCTCCTGGAAGATGGTGAT-3′, TLR4:F 5′-TGAGCAGTCGTGCTGGTATC-3′ R 5′-CAGGGCTTTTCTGAGTCGTC-3′, NF-κB: F 5′-CAATGCCCTTTTCGACTACGC-3′ R 5′-AGCCCTCAGCAAATCCTCCA-3′. All experiments were performed in triplicate.

### Western blot

Collected macrophages were lysed in cold RIPA buffer (Beyotime, China) supplemented with protease inhibitors (Beyotime, China) and phosphatase inhibitors (Beyotime, China). Protein concentration was then quantified using Pierce BCA Protein Assay Kit (Beyotime, China). Proteins were separated in polyacrylamide gel and transferred to the PVDF membranes(Biosharp, China). After blocked, membranes were separately incuba- ted with anti-TLR4(Beyotime, China), anti-IκBα (Beyotime, China), anti-p-IκBα(Beyotime,China), anti-NFκB(Beyotime,China),anti-p-NFκB(Beyotime, China)antibodies at 4 °C overnight. After washing with TBST, membranes were incubated with HRP-conjugated anti-rabbit IgG (Beyotime, China). The blots were visualized with an Enhanced Chemiluminescence Kit (NCM, China). The level of GAPDH (Beyotime, China) was used as the internal standard. All experiments were performed in triplicate.

### ELISA

Quantitative detection of TNF-α, IL-6 and IL-1β in macrophage culture supernatant was performed using ELISA detection kit (MULTISCENCES, China), and the operation was performed according to the instructions for use, and the concentration was finally calculated according to the OD value. All experiments were performed in triplicate.

### Animal model of endometrial injury

All animal experiment procedures were conducted according to the National Institutes of Health guide for the care and use of Laboratory animals (NIH Publications No. 8023, revised 1978), were approved by the ethics committee of Shanghai Tongji Hospital(2022-DW-(006)). Female Sprague-Dawley(SD) rats(180–200 g, 6–8weeks old) were cycle synchronized by vaginal smear analysis, rats in proliferative phase were used for the severe endometrium injury model as previse described [17]. In brief, after anesthesia, the first incision was made in the abdominal wall, then a severe uterus injury was made by scraping endometrium completely using a scalpel blade for a segment of approximately 1.5 cm in length and 0.3 cm in width from each uterine horn (Supplementary Fig. 2). Rats were divided into ①Fg/P(LLA-CL) group (9 rats in total): Fg/P(LLA-CL) film (1.5 cm × 0.3 cm) was placed on the uterine injury site and the incision was closed with 7–0 silk suture (Johnson& Johnson, USA). ②Control group (9 rats in total): rats with severe endometrium damage received no treatment, the uterine incision was sutured directly. Three rats in each group were sacrificed 3/7/14 days after operation, and the uterus was removed and fixed for immunofluorescence.

### RNA-sequencing

To figure out the transcriptome differences between the Fg/P(LLA-CL) group and the Control group, we performed RNA-sequencing of 3 rat uterus each group 14 days after surgery. RNA-seq were performed by Personal Biotechnology Co., Ltd. (Shanghai, China). We used R software to process the sequencing data, differentially expressed genes were calculated and filtered by *p* < 0.05 and fold-change > 2(using the “limma” package). Gene Ontology(Go) functional enrichment analysis for differentially expressed genes were conducted by R package “clusterProfiler”.

### Immunofluorescence

The rat uterine tissue samples were routinely processed for immunohistochemical analysis. They were embedded in paraffin, sectioned, dewaxed, and blocked. Subsequently, diluted antibodies against CD68 (Abcam, USA), CD163 (Servicebio, China), CD86 (Beyotime, China), CD90 (Servicebio, China), CD105 (Proteintech, China), and CD14 (Servicebio, China) were applied to the sections. The sections were incubated overnight in the dark and then washed three times with PBS for 5 min each. Next, the tissues were covered with appropriate species-specific HRP secondary antibodies (HRP-labeled Goat Anti-Rabbit IgG, HRP-labeled Goat Anti-Mouse IgG, Beyotime, China) and incubated in the dark at room temperature for 50 min. TYR fluorescent dyes (ABclonal, China) with distinct emission wavelengths (green: Ex/Em = 490/520, red: Ex/Em = 550/570) were added dropwise for 10 to 15 min. The sections were then removed, warmed at 37 °C for 45 min, and rinsed three times with PBS for 5 min each. After slight drying, DAPI staining solution (Servicebio, China) was applied and incubated for 10 min in the dark at room temperature. The sections were washed with PBS again, dried, mounted with anti-fluorescence quenching mounting agent, and observed under a fluorescence microscope. For multiple-label immunofluorescence staining, after observation under the fluorescence microscope, the antibodies on the sections were stripped using an antibody stripping solution. The incubation steps with the primary antibodies, secondary antibodies, and TYR dyes were then repeated.

### Statistic analysis

ImageJ software (U.S. Pat. National Institutes of Health, MD, USA) was used to perform immunofluorescence. Statistical analysis was performed using GraphPad Prism software (San Diego, CA, USA, version 9) and all data represent the mean ± standard deviation (SD) of three independent experiments. The comparison between the two groups was carried out by student *t* test, statistical significance was considered for *p < 0.05; **p < 0.01; ***p < 0.001; ****p < 0.0001.

## Results

### Electron microscopic morphology of Fg/P(LLA-CL)

**Fig. 1:**
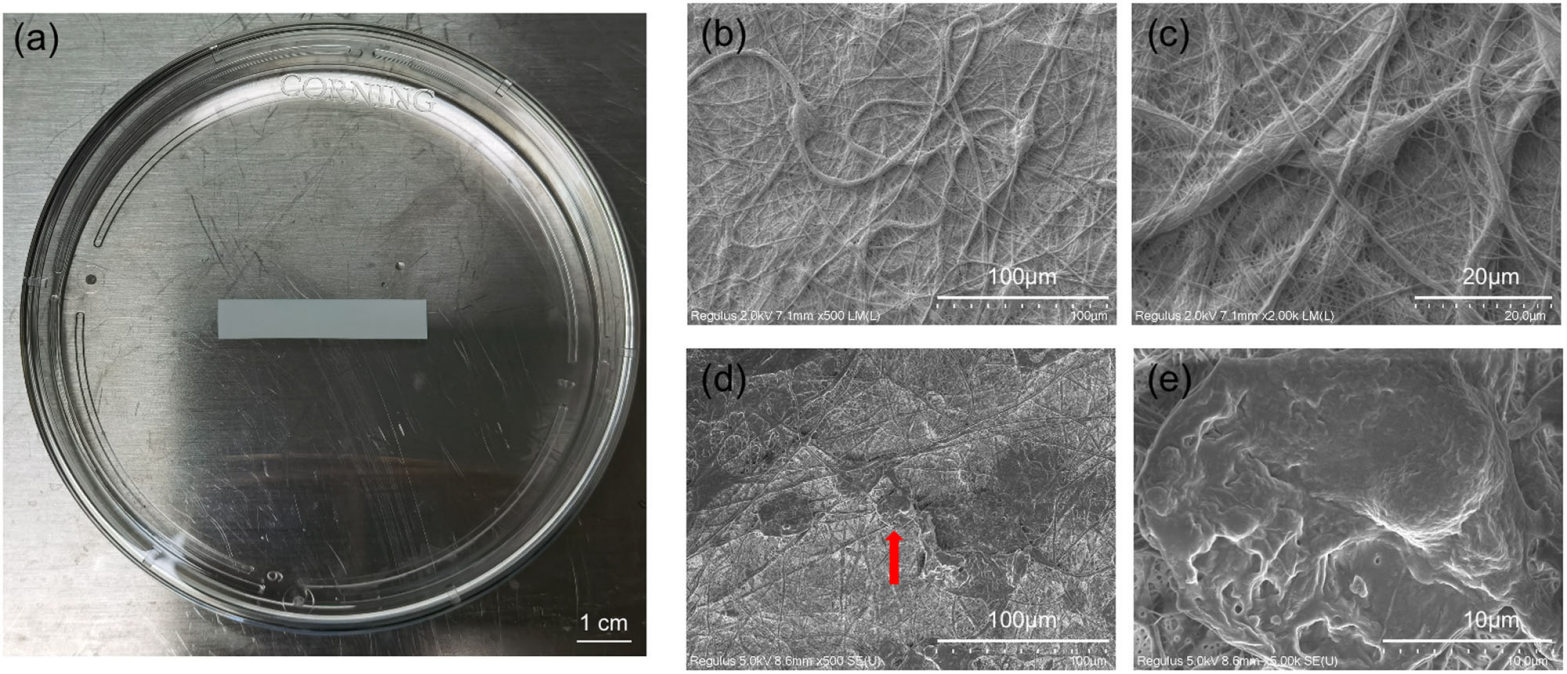
Appearance of Fg/P(LLA-CL) (**a**). Representative SEM micrographs showing microstructure of Fg/P(LLA-CL) (**b**, **c**). Rat adipose-derived mesenchymal stem cells (red arrow) adhere to Fg/P(LLA-CL) (**d**, **e**)

As depicted in Fig. 1a, we specifically designed the Fg/P(LLA-CL) nanomaterial to conform to the dimensions of the rat uterus. During the surgical procedure, it was trimmed to a size of 1.5 × 0.3 cm and implanted into the uterine cavity of the rats. Examination under an electron microscope revealed that the majority of the fibers within the Fg/P(LLA-CL) construct exhibited diameters ranging from 100 to 400 nanometers (Fig. 1b, c). Furthermore, it was observed that the ADSCs were able to adhere effectively to the Fg/P(LLA-CL) surface (Fig. 1d, e).

### RNA-seq of rat uterus

**Fig. 2:**
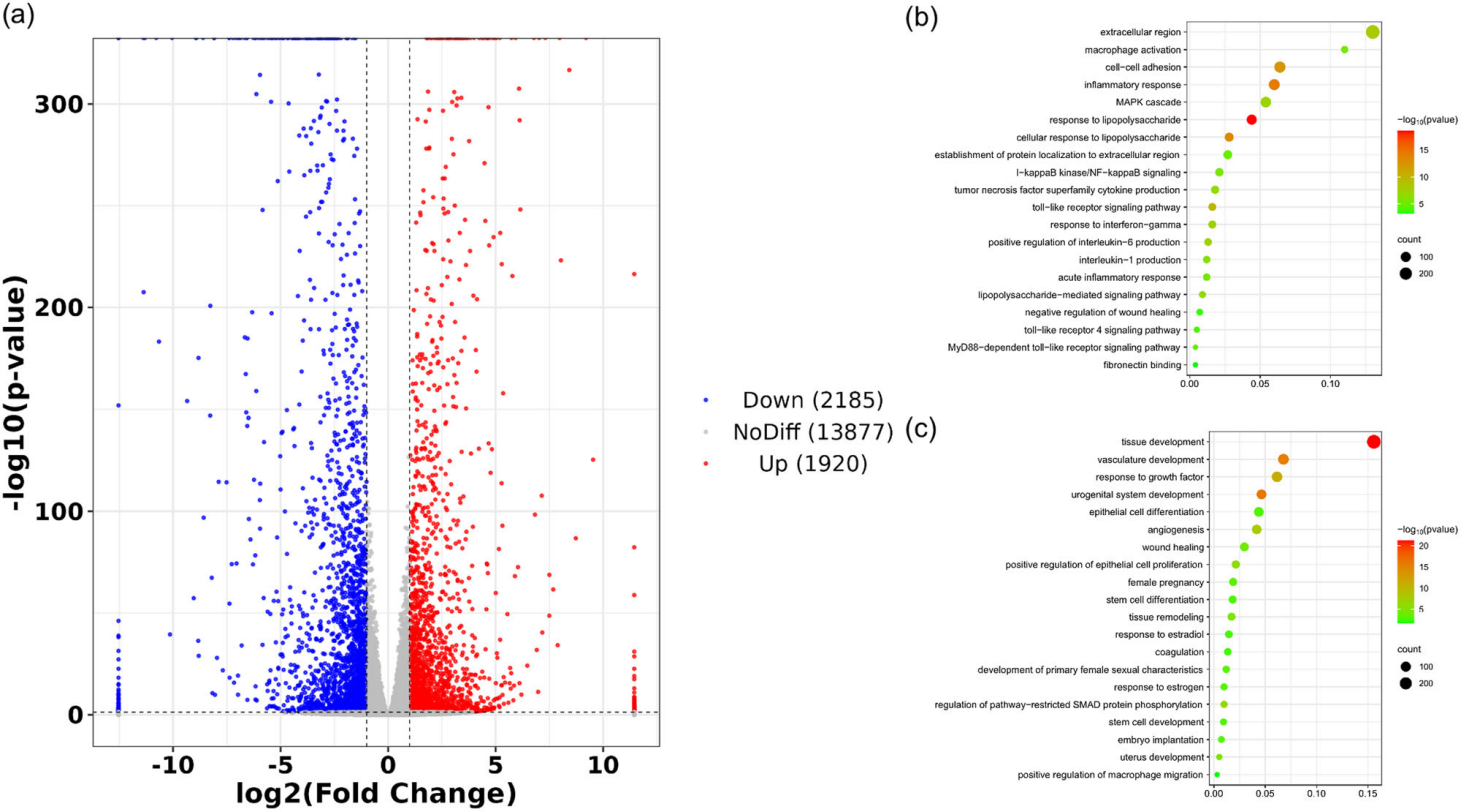
Differentially expressed genes between Fg/P(LLA-CL) and Con group (**a**). Representative GO terms for downregulated genes (**b**). Representative GO terms for upregulated genes (**c**)

Histological morphology of rat uterus after endometrial injury was shown in Supplementary Fig. 3. Notably, the uterine cavity of the control group closed completely at 3 and 7 days post-operation, with intrauterine adhesion and hydrops observed at 14 days. Conversely, endometrial regeneration was observed in the uterus of rats treated with Fg/P(LLA-CL). To further investigate, RNA-sequencing was performed on the recovering uterus at 14 days post-surgery. The results revealed that 1920 genes were up-regulated and 2185 genes were down-regulated in the Fg/P(LLA-CL) group compared to the control (Fig. 2a). GO enrichment analysis indicated that the down-regulated genes were associated with TLR4/NFκB signaling, lipoprotein response, and macrophage activation, while the up-regulated genes were related to coagulation, stem cell differentiation, and stem cell development (Fig. 2b, c). Based on these findings, we hypothesize that Fg/P(LLA-CL) may regulate macrophage phenotype via the TLR4/NFκB signaling pathway and influence the biological behavior of stem cells following endometrial injury.

### Fg/P(LLA-CL) prevents macrophage polarization to M1 phenotype through TLR4/NFκB signaling pathway

**Fig. 3:**
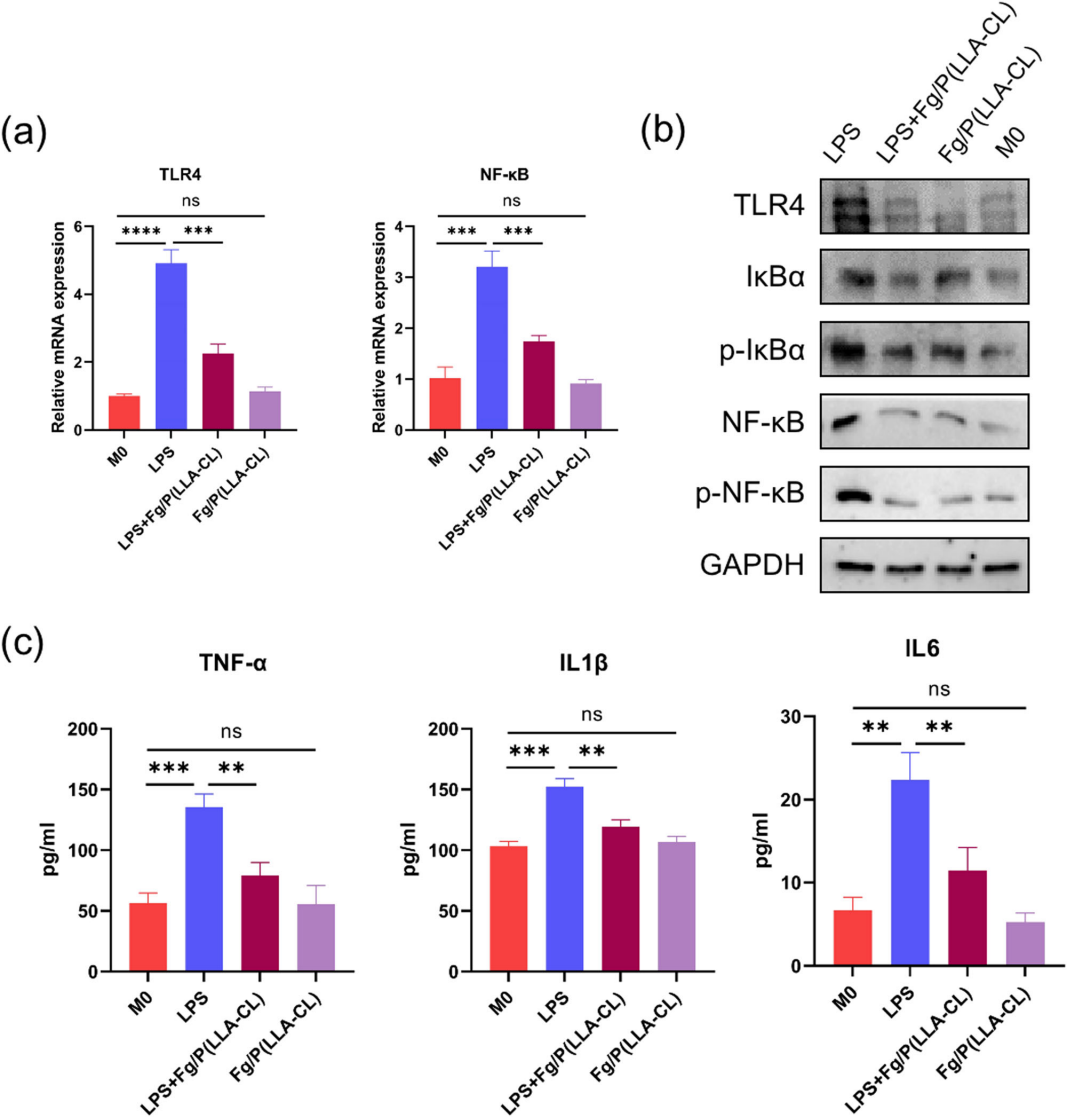
The expression of TLR4 and NF-κB in different groups was detected by qPCR (**a**). Western blot was used to detect the expression of TLR4/NF-κB signaling pathway related proteins in different groups (**b**). The concentrations of TNF-α, IL-1β and IL-6 in the conditioned medium of macrophages were detected by ELISA (**c**). (*n* = 3)

LPS, an activator of the macrophage TLR4/NFκB signaling pathway, was utilized to simulate inflammatory stimulation. Our qPCR and Western Blot experiments revealed that Fg/P(LLA-CL) alone did not alter the TLR4/NFκB signaling state in macrophages. However, upon LPS stimulation, this pathway became activated, and Fg/P(LLA-CL) effectively inhibited this activation (Fig. 3a, b). Furthermore, ELISA analysis demonstrated that LPS stimulation increased the proinflammatory cytokines TNF-α, IL-1β, and IL-6, cytokines associated with the M1 phenotype of macrophages. Notably, Fg/P(LLA-CL) suppressed the secretion of these inflammatory factors (Fig. 3c).

**Fig. 4:**
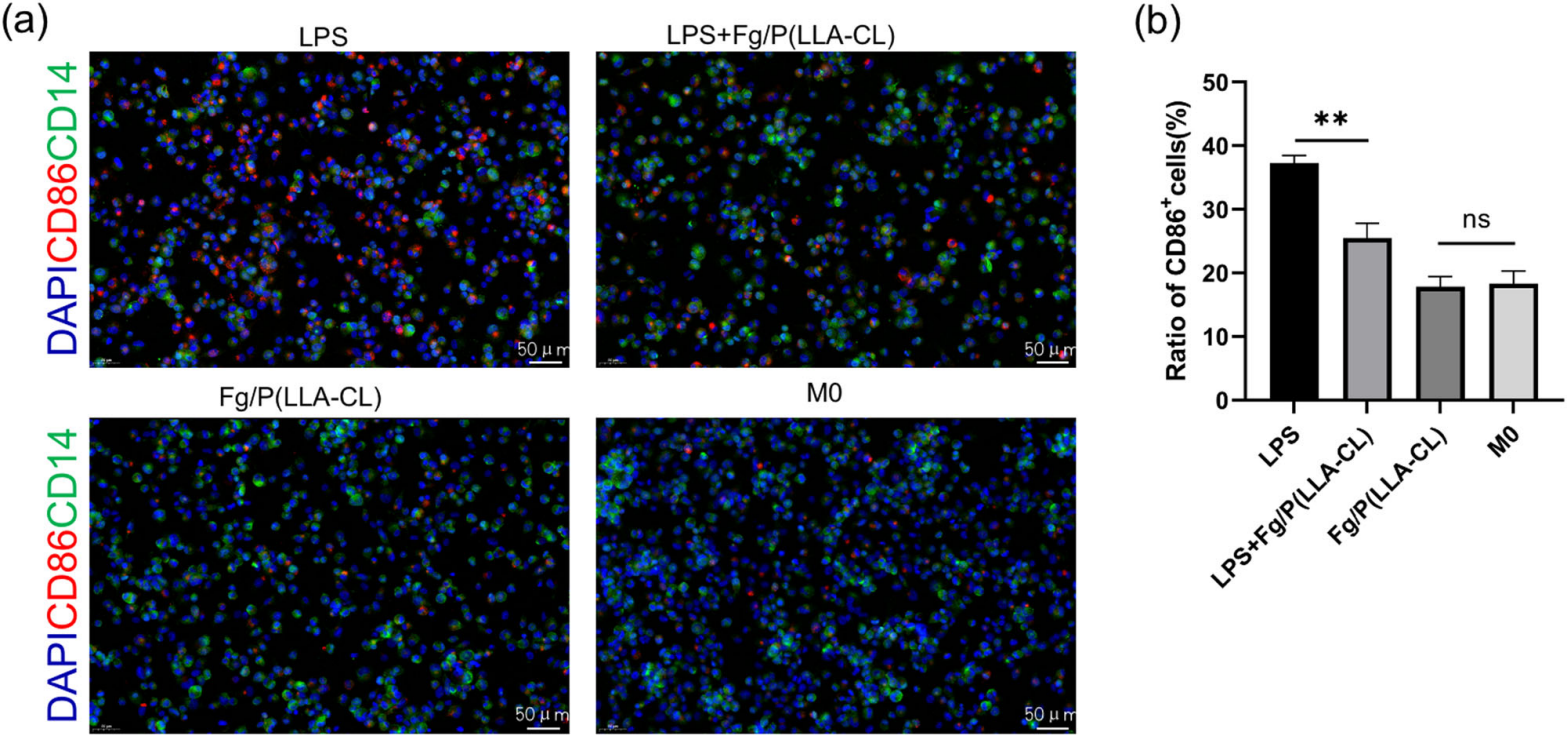
The expression of the macrophage M1 phenotype surface marker CD86 in different groups was observed by immunofluorescence in different groups (**a**). Proportion of CD86+ cells in different groups (**b**). (*n* = 3)

Utilizing immunofluorescence to analyze the surface marker CD86 of M1 macrophages, we confirmed that Fg/P(LLA-CL) effectively suppressed the polarization of macrophages towards the M1 phenotype following LPS stimulation. Notably, Fg/P(LLA-CL) alone did not alter the polarization state of macrophages (Fig. 4).

### Fg/P(LLA-CL) alters macrophage phenotype in vivo

**Fig. 5:**
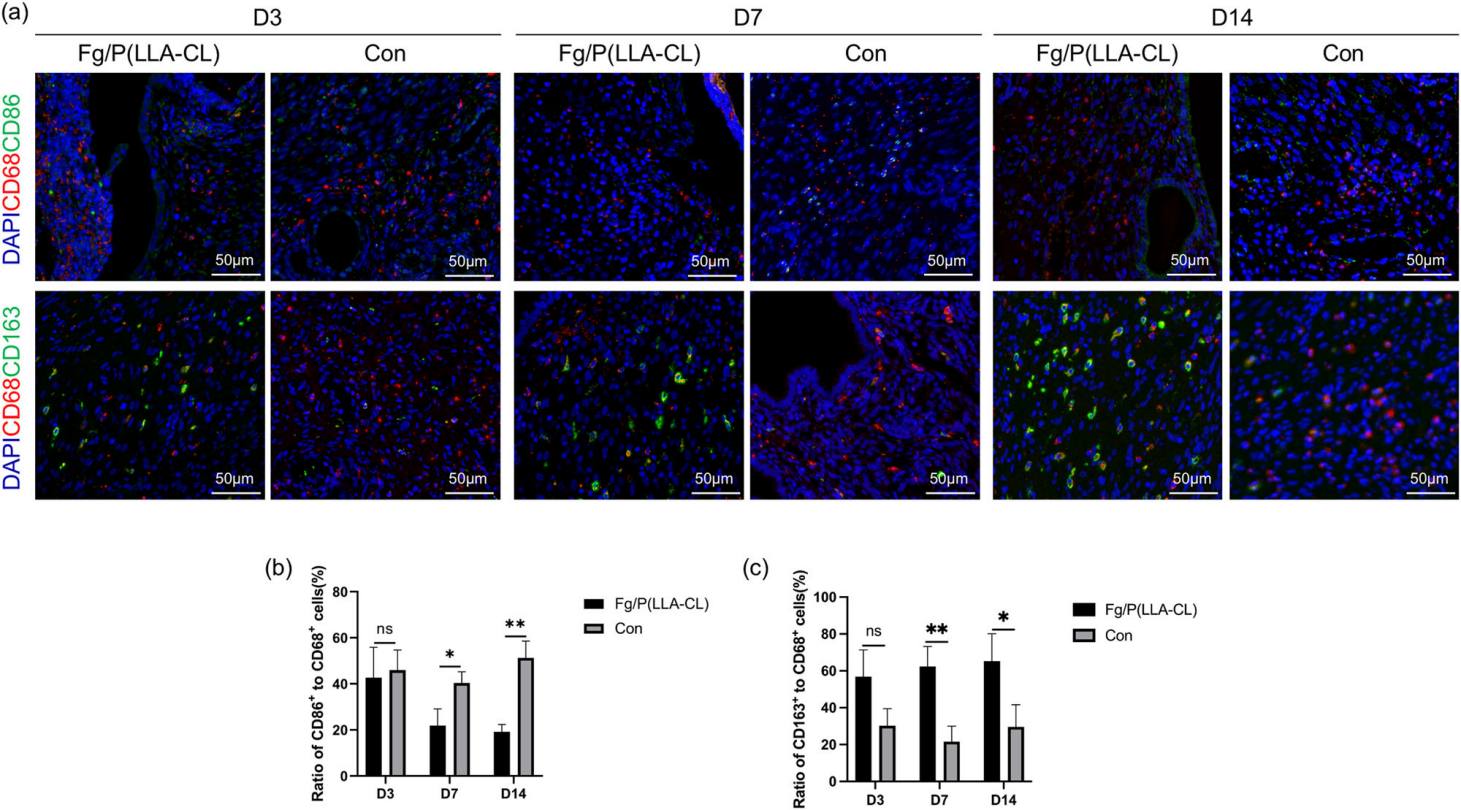
Immunofluorescence was used to observe the expression of macrophage M1 phenotype surface marker CD86 and M2 phenotype surface marker CD163 in Fg/P(LLA-CL) group and control group (**a**). Proportion of macrophages with M1 phenotype (**b**) and M2 phenotype (**c**) at 3 days, 7 days and 14 days after operation in different groups. (*n* = 3)

Immunofluorescence analysis of uterine tissue revealed that at 3 days post-operation, CD86 expression on M1 macrophages in the Fg/P(LLA-CL) group resembled that of the Control group. While CD163 expression, a marker for M2 macrophages, was higher in the Fg/P(LLA-CL) group, this difference was not statistically significant. However, at 7 and 14 days post-operation, CD163 expression was significantly higher in the Fg/P(LLA-CL) group, whereas CD86 expression was lower. These findings suggest that Fg/P(LLA-CL) promotes macrophage polarization towards the anti-inflammatory M2 phenotype and suppresses the pro-inflammatory M1 phenotype (Fig. 5).

### Fg/P(LLA-CL) recruits mesenchymal stem cells(MSCs) in vivo

**Fig. 6:**
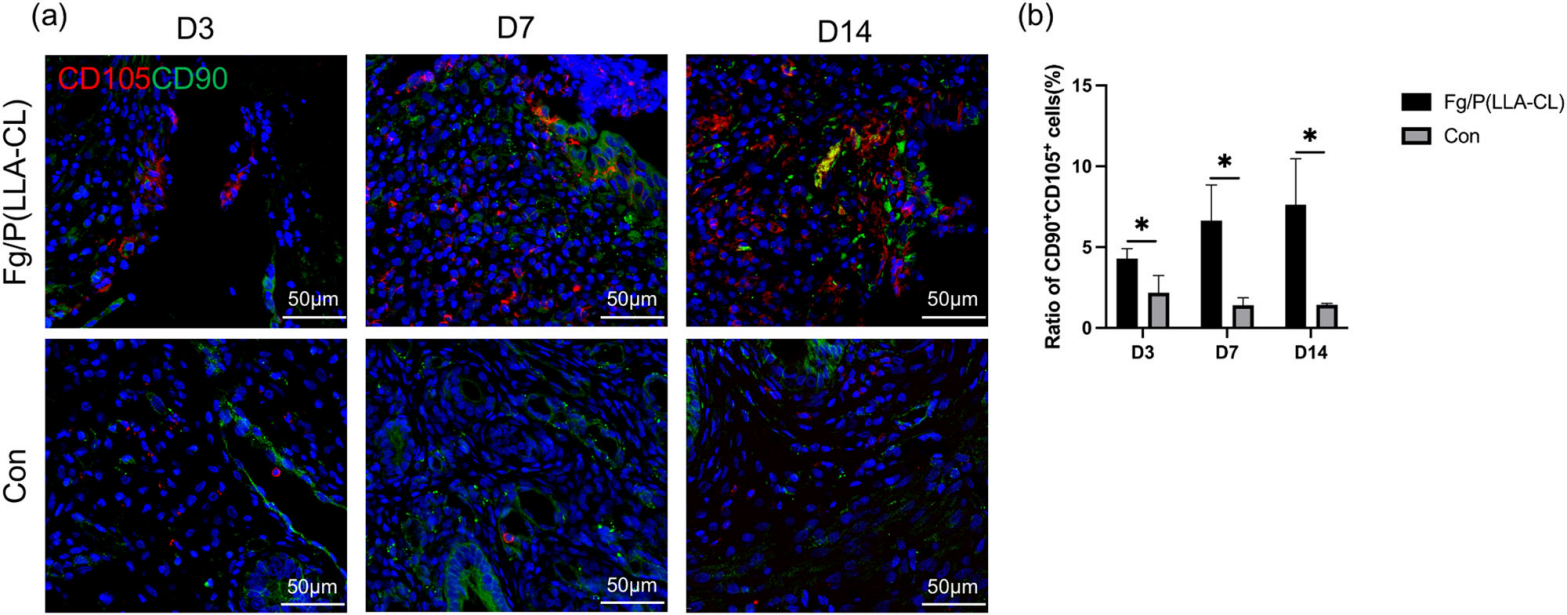
Immunofluorescence was used to observe the expression of mesenchymal stem cell surface markers CD105^+^CD90^+^ in the injured site of rat endometrium (**a**). Comparison of the number of mesenchymal stem cells in different groups at 3, 7 and 14 days after operation (**b**). (*n* = 3)

In our GO analysis, genes upregulated in the Fg/P(LLA-CL) group were enriched in stem cell differentiation and development. To investigate the effect of Fg/P(LLA-CL) on MSCs, we labeled MSC surface markers CD90 and CD105 via immunofluorescence. Our results showed a higher number of MSCs at the lesion site in the Fg/P(LLA-CL) group compared to the control group at 3, 7, and 14 days post-endometrial injury (Fig. 6). This confirmed the in vivo migration of MSCs to the injured site under the influence of Fg/P(LLA-CL).

## Discussion

Biomaterials are widely utilized in tissue repair, with the modulation of the immune microenvironment in damaged tissue serving as a crucial mechanism for their functional efficacy [18]. The modulation of macrophage polarization and function represents an effective approach for biomaterials to mitigate fibrosis following tissue injury [19].

Fibrinogen is the initial protein essential following tissue injury. During coagulation, thrombin converts fibrinogen into fibrin, which forms a three-dimensional network in the clot, thereby prolonging the retention of cytokines pertinent to tissue repair [20]. Furthermore, the integration of fibrinogen with biomaterials enhances cell adhesion, proliferation, and differentiation, while also modulating immune cell function [21]. For instance, Simaan-Yameen et al. successfully combined fibrinogen with methacrylation to create FibMA hydrogel, an excellent 3D cell culture medium [22]. Lu et al. demonstrated that coating Carbon nanotubes with fibrinogen reduced their cytotoxicity towards macrophages [23]. Cao H discovered that calcium-doped titanium alone lacks antibacterial activity, but its adsorption of fibrinogen strongly inhibits bacterial colonization [24]. In our previous study, we have conducted a detailed examination of the physicochemical properties and biocompatibility of Fg/P(LLA-CL), The fiber diameter of Fg/P(LLA-CL) is approximately 329 ± 12 nm, with a water contact angle of 66 ± 3° and a porosity of 50.2 ± 1.6% [16]. In this study, we demonstrated that Fg/P(LLA-CL) can regulate macrophage phenotype and function.

LPS stimulation activates the TLR4/NFκb signaling pathway in macrophages, crucial in chronic intrauterine inflammation and adhesions due to the inevitable presence of non-sterile conditions during intrauterine procedures [25, 26]. Some studies have reported Fg’s activation of the NFκB signaling pathway in monocytes [27]. However, consistent with Mafalda Bessa-Gonçalves et al. [10, 28] our findings indicate that Fg/P(LLA-CL) alone does not activate TLR4/NFκb signaling. Instead, it inhibits LPS-induced TLR4/NFκb activation, macrophage polarization towards the M1 phenotype, and the secretion of inflammatory cytokines, including IL-1β, IL-6, and TNF-α. Macrophages in vivo can be categorized as tissue-resident or derived from circulating monocytes. Following tissue injury, these macrophages migrate towards the injury site and polarize in diverse directions, influenced by the specific tissue microenvironment [29], this observation may account for the lack of significant difference in the proportion of pro- and anti-inflammatory macrophages between the Fg/P(LLA-CL) and control groups at 3 days postoperatively. However, macrophages in the Fg/P(LLA-CL) group exhibited a significant shift towards an anti-inflammatory phenotype at 7 and 14 days postoperatively. Both IL-1β and TNF-α have been proved to be highly expressed in intrauterine adhesions, which act on fibroblasts to synthesize a large amount of collagen and lead to excessive deposition of ECM to form intrauterine adhesions [30]. In addition, the high expression of IL-1β and TNF-α is also an important inducement of fibrosis in liver, skin, peritoneum and lung [31–33]. Therefore, we have reason to believe that Fg/P(LLA-CL) can reduce tissue inflammation by inhibiting macrophages to pro-inflammatory phenotype, thus playing a positive role in the prevention of intrauterine adhesions after endometrial injury.

After intrauterine procedures, damage to the endometrial basal layer and loss of endometrial progenitor cells/mesenchymal stem cells are key contributors to intrauterine adhesions [34]. Stem cell augmentation has emerged as a pivotal research avenue for intrauterine adhesions, exhibiting efficacy in numerous animal and clinical trials. Nevertheless, exogenous stem cells pose numerous challenges, including stringent culture requirements, loss of stemness post-culture, substantial costs, and ethical considerations [35–37]. After tissue injury, macrophages secrete chemokines to attract stem or progenitor cells. However, in the hypoxic microenvironment post-injury, chemokine secretion is frequently inadequate [38]. With some biomaterials, macrophages can dramatically increase the number of stem cells recruited to the site of injury [39, 40]. After intrauterine implantation of Fg/P(LLA-CL), we observed enhanced recruitment of MSCs to endometrial injury sites. MSCs have demonstrated therapeutic potential in various diseases, including liver injury, pulmonary fibrosis, and myocardial injury, by promoting tissue repair and reducing fibrosis. However, elucidating the mechanism underlying their therapeutic effects, whether through directional differentiation or paracrine effects, and identifying the specific paracrine factors that promote tissue repair and reduce fibrosis, remains challenging [41–43]. Our study revealed that Fg/P(LLA-CL) modulates macrophage polarization in inflammatory environments and enhances MSC recruitment to injury sites. Future work involves optimizing the Fg:P(LLA-CL) ratio to maximize the biomaterial’s effects. Additionally, we aim to explore the role of macrophages in stem cell recruitment and conduct further animal experiments to assess stem cell recruitment in the absence of macrophages. Finally, we seek to delve deeper into the mechanisms of mesenchymal stem cells’ therapeutic actions at injury sites.

## Conclusion

For the first time, we have shown that Fg/P(LLA-CL) inhibits LPS-induced M1 macrophage polarization in vitro and modulates macrophage polarization in an endometrial injury animal model. Additionally, this nano-biomaterial promotes MSC recruitment to injury sites, suggesting its potential for further investigation and application in intrauterine adhesions and other tissue injuries.

## Supplementary information

Supplementary Figure 1

Supplementary Figure 2

Supplementary Figure 3

Supplementary Figure legends
