# Supplementary figures and images for: Biomaterial Fg/P(LLA-CL) regulates macrophage polarization and recruitment of mesenchymal stem cells after endometrial injury
Source: J Mater Sci Mater Med. 2024 Jul 29;35(1):39. doi: 10.1007/s10856-024-06807-w (PMC11286705; doi:10.1007/s10856-024-06807-w)

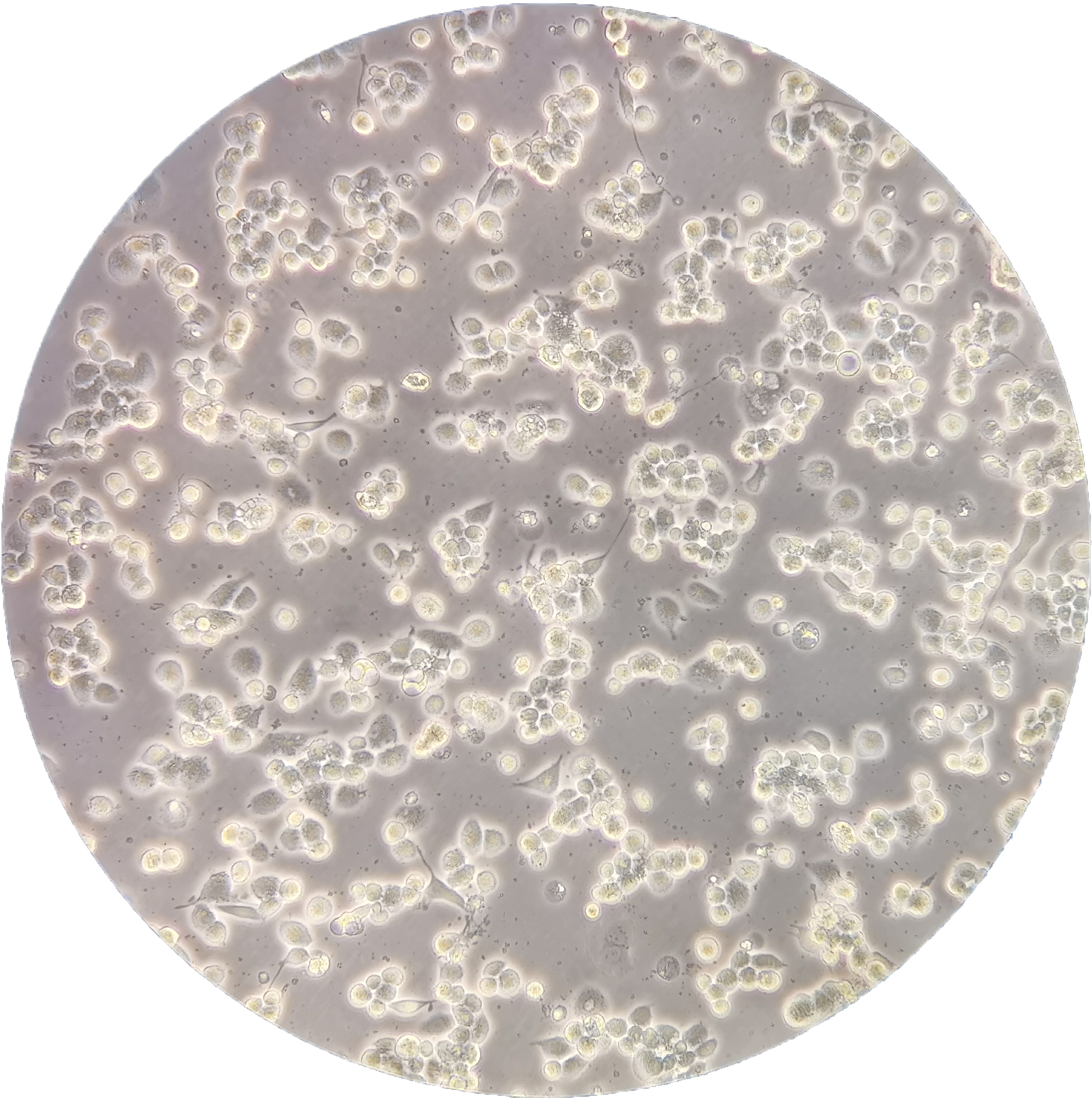

Supplement: Supplementary file 1 — Supplementary Figure 1 [file 10856_2024_6807_MOESM1_ESM.tif]

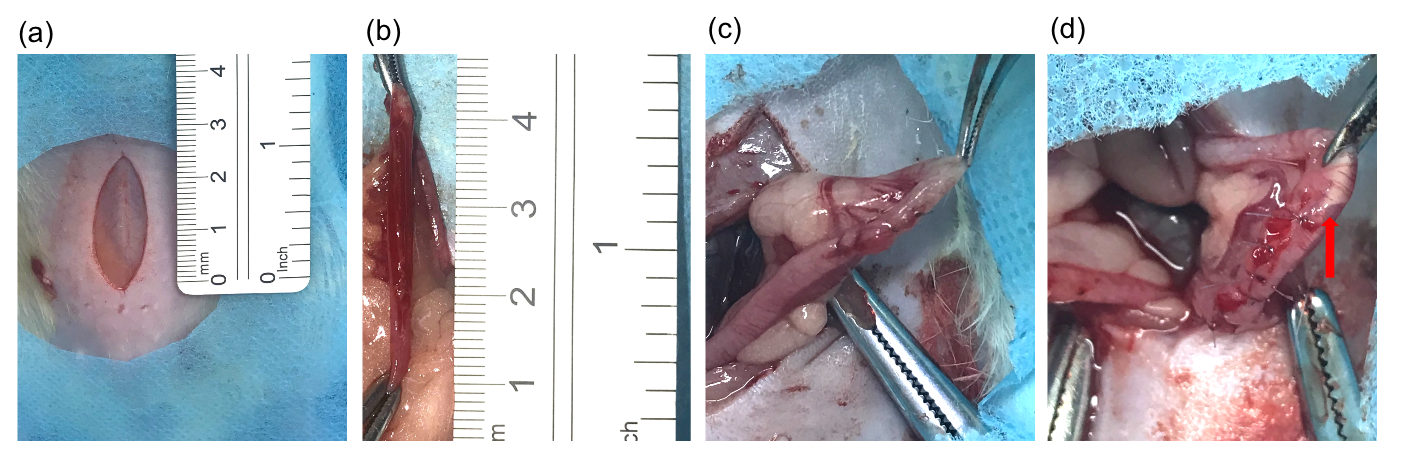

Supplement: Supplementary file 2 — Supplementary Figure 2 [file 10856_2024_6807_MOESM2_ESM.tif]

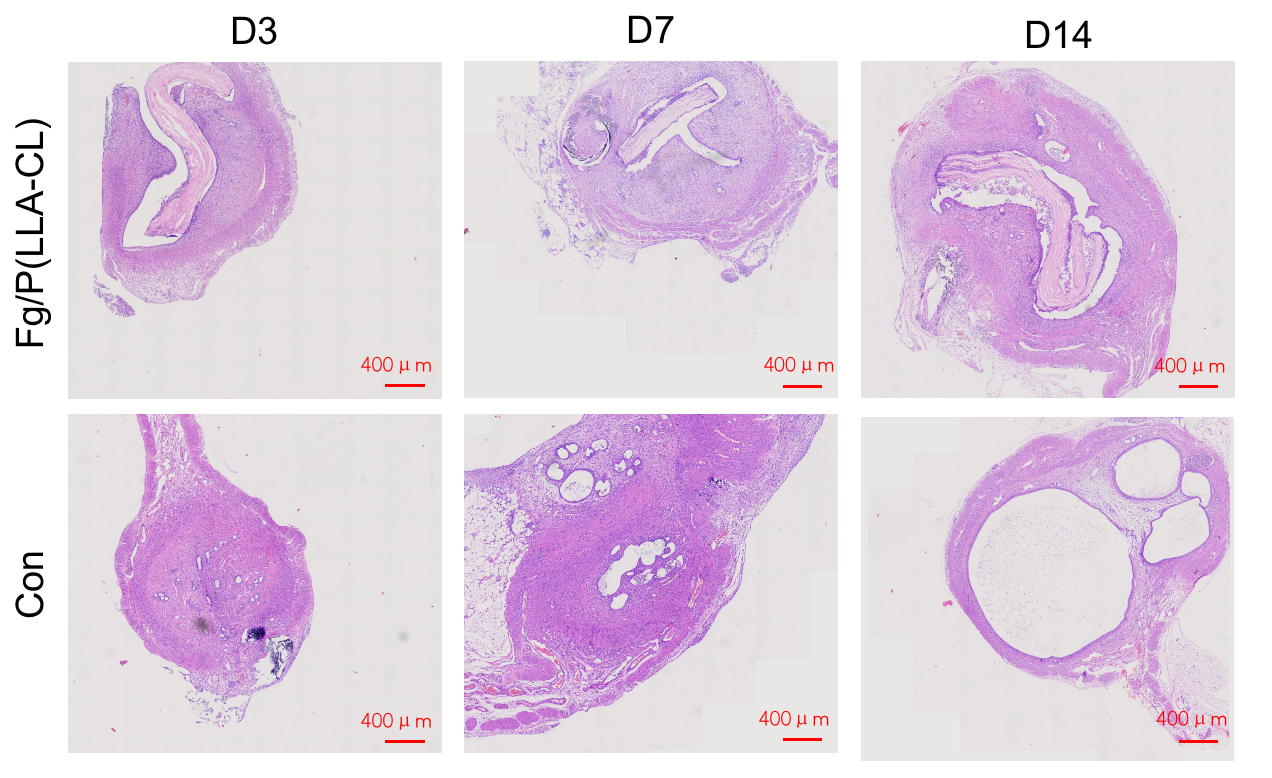

Supplement: Supplementary file 3 — Supplementary Figure 3 [file 10856_2024_6807_MOESM3_ESM.tif]
