# Supplementary material for: Biomaterial Fg/P(LLA-CL) regulates macrophage polarization and recruitment of mesenchymal stem cells after endometrial injury
Source: J Mater Sci Mater Med. 2024 Jul 29;35(1):39. doi: 10.1007/s10856-024-06807-w (PMC11286705; doi:10.1007/s10856-024-06807-w)
Supplement: Supplementary file 4 — Supplementary Figure legends [file 10856_2024_6807_MOESM4_ESM.docx]

Supplement figure 1. Macrophages induced from THP-1 cells were observed under microscope(10X).

Supplement figure 2. Surgical procedure. Make an incision on the abdomen(a). Find the uterus and destroy the endometrium(b). Suture the uterus without Fg/P(LLA-CL)(c). The uterus was sutured after Fg/P(LLA-CL) (red arrow)was placed in the uterine cavity(d).

Supplement figure 3. Histological morphology of rat uterus in different groups at 3, 7 and 14 days after endometrial injury.
